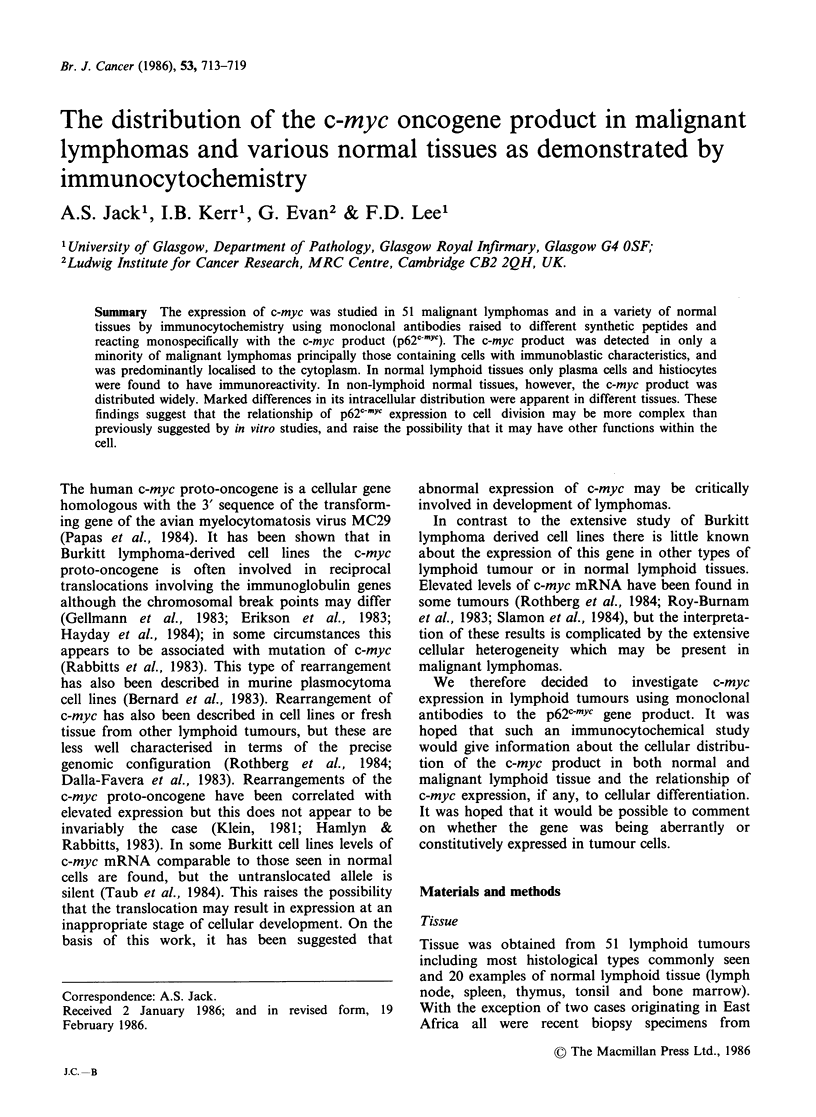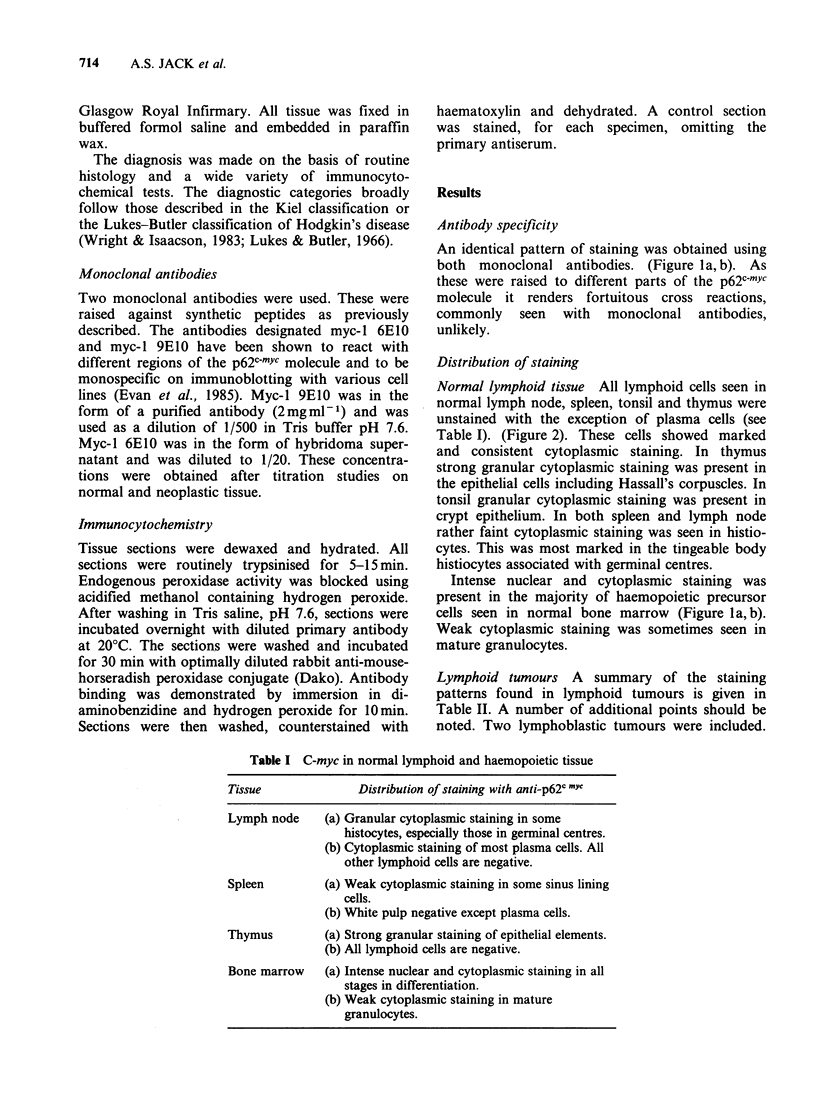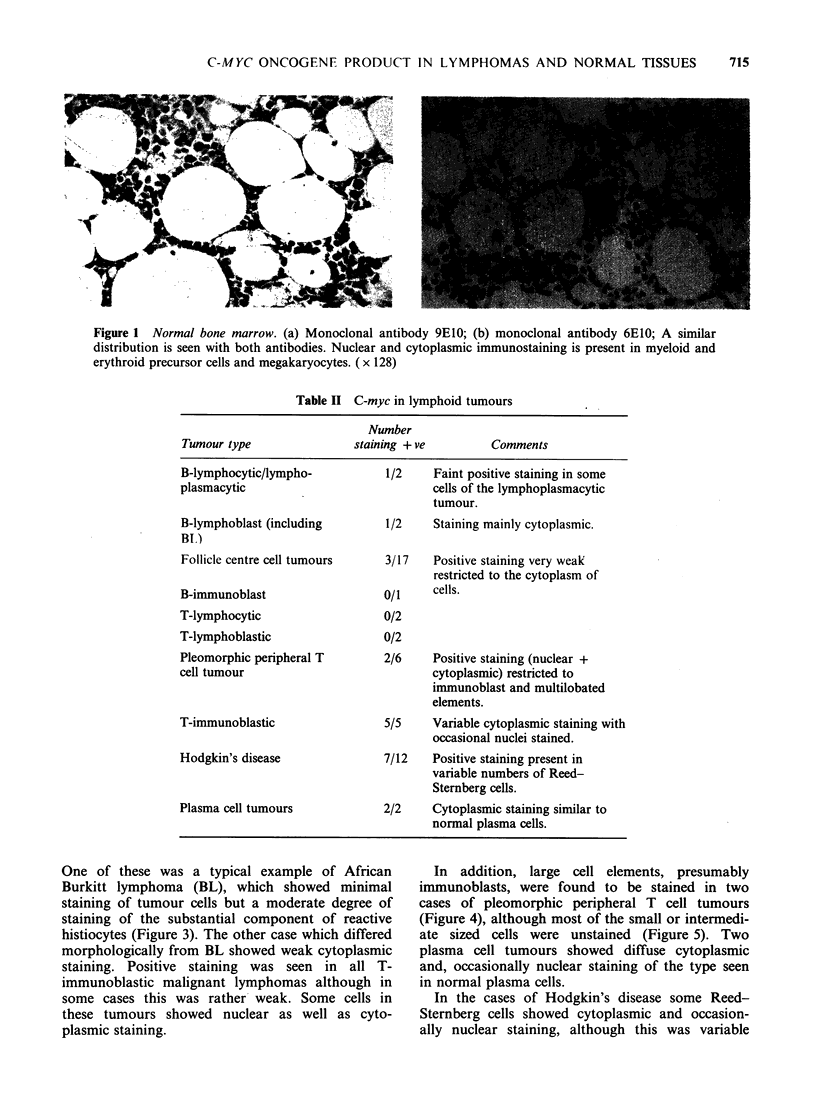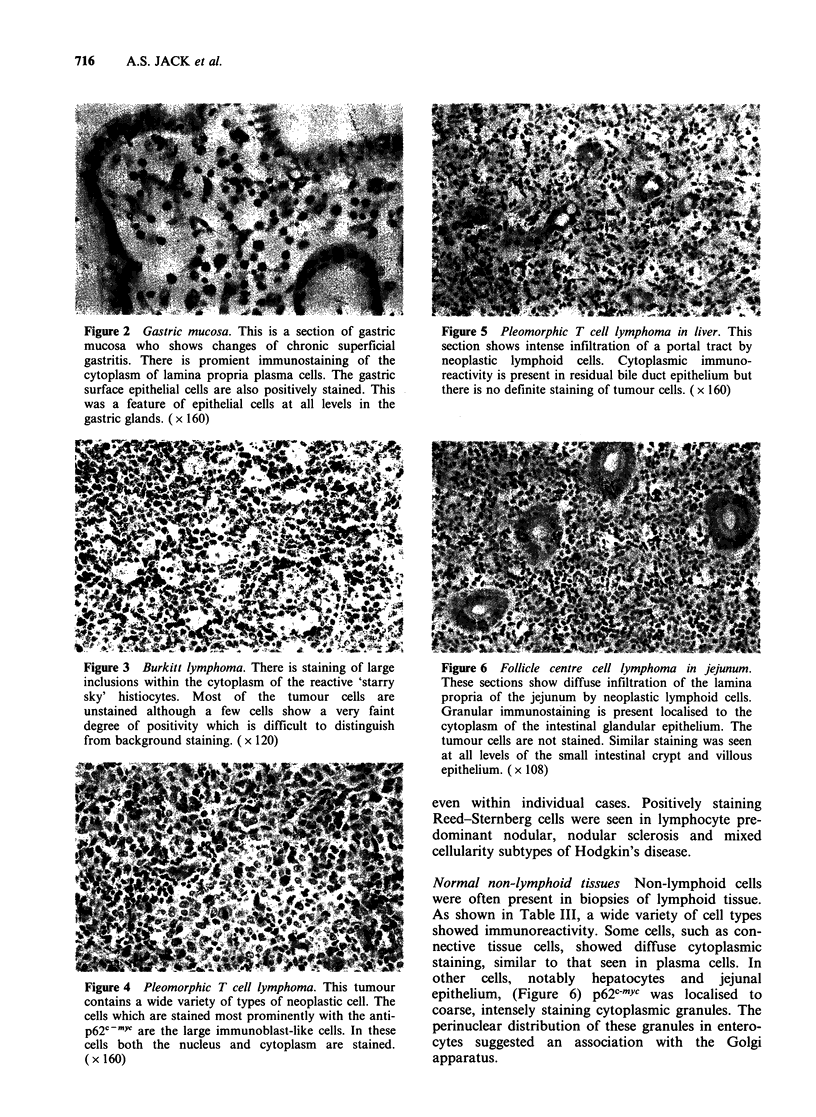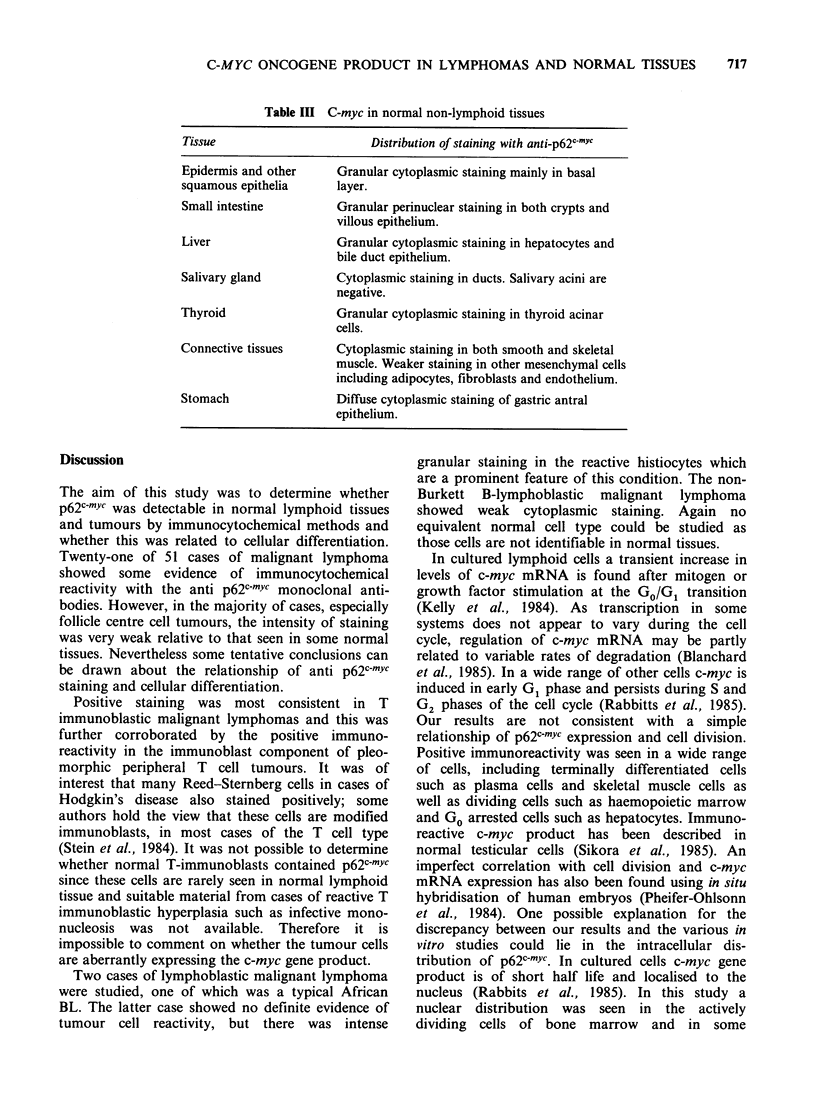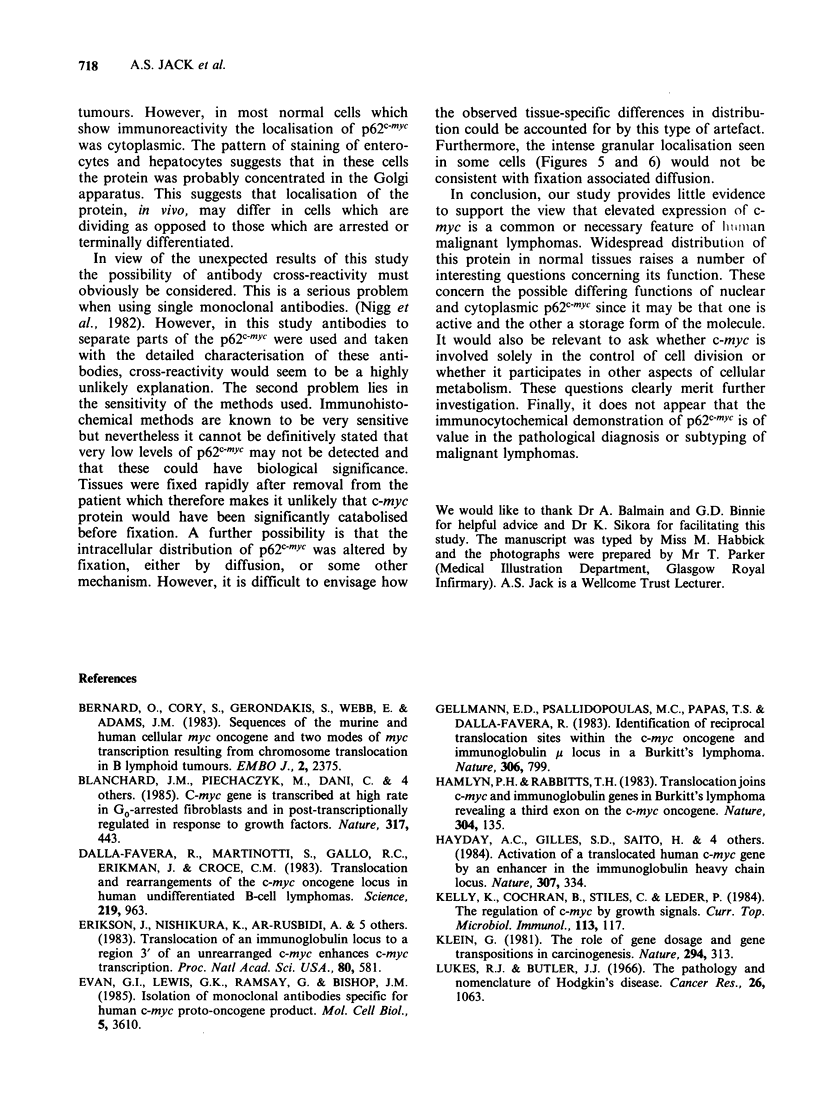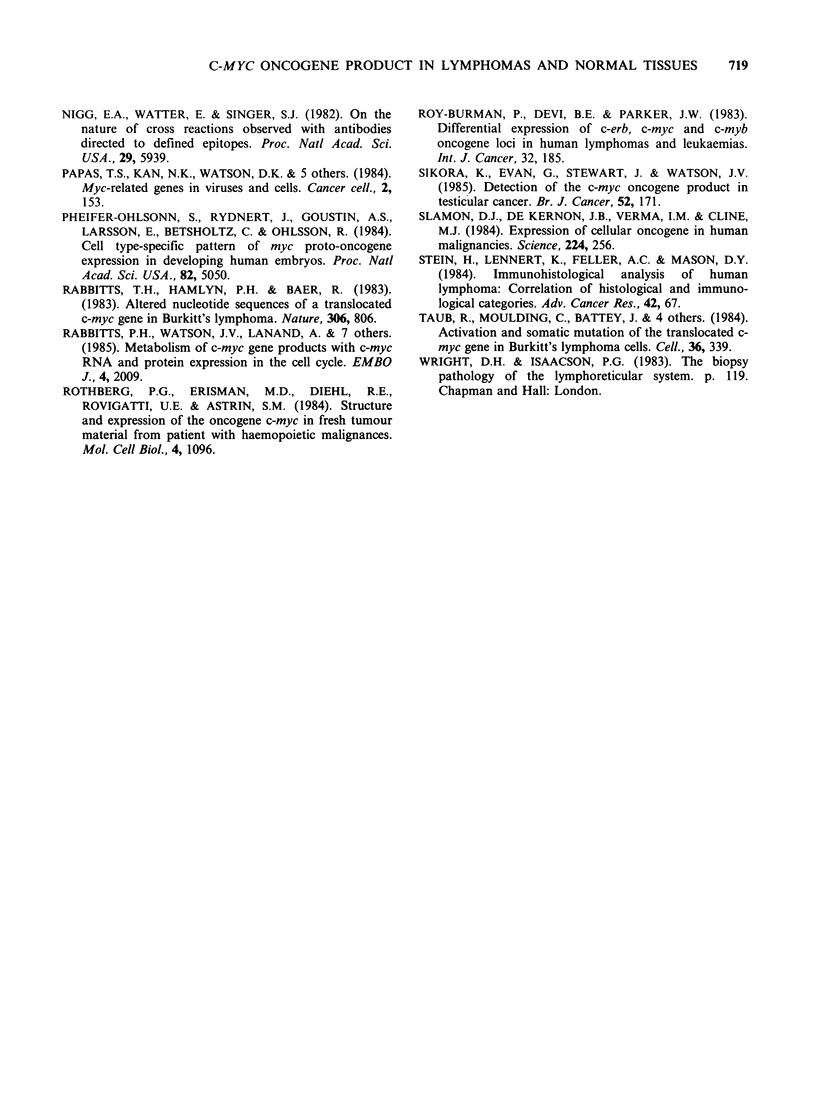# The distribution of the c-myc oncogene product in malignant lymphomas and various normal tissues as demonstrated by immunocytochemistry.

**DOI:** 10.1038/bjc.1986.123

**Published:** 1986-06

**Authors:** A. S. Jack, I. B. Kerr, G. Evan, F. D. Lee

## Abstract

**Images:**


*Br. J. Cancer* (1986), **53**, 713–719

### The distribution of the c-*myc* oncogene product in malignant lymphomas and various normal tissues as demonstrated by immunocytochemistry

A.S. Jack[1], I.B. Kerr[1], G. Evan[2] & F.D. Lee[1]

[1]*University of Glasgow, Department of Pathology, Glasgow Royal Infirmary, Glasgow G4 0SF;*
[2]*Ludwig Institute for Cancer Research, MRC Centre, Cambridge CB2 2QH, UK.*

**Summary** The expression of c-*myc* was studied in 51 malignant lymphomas and in a variety of normal tissues by immunocytochemistry using monoclonal antibodies raised to different synthetic peptides and reacting monospecifically with the c-*myc* product ($p62^{c\text{-}myc}$). The c-*myc* product was detected in only a minority of malignant lymphomas principally those containing cells with immunoblastic characteristics, and was predominantly localised to the cytoplasm. In normal lymphoid tissues only plasma cells and histiocytes were found to have immunoreactivity. In non-lymphoid normal tissues, however, the c-*myc* product was distributed widely. Marked differences in its intracellular distribution were apparent in different tissues. These findings suggest that the relationship of $p62^{c\text{-}myc}$ expression to cell division may be more complex than previously suggested by *in vitro* studies, and raise the possibility that it may have other functions within the cell.

The human c-*myc* proto-oncogene is a cellular gene homologous with the 3′ sequence of the transforming gene of the avian myelocytomatosis virus MC29 (Papas *et al.*, 1984). It has been shown that in Burkitt lymphoma-derived cell lines the c-*myc* proto-oncogene is often involved in reciprocal translocations involving the immunoglobulin genes although the chromosomal break points may differ (Gellmann *et al.*, 1983; Erikson *et al.*, 1983; Hayday *et al.*, 1984); in some circumstances this appears to be associated with mutation of c-*myc* (Rabbitts *et al.*, 1983). This type of rearrangement has also been described in murine plasmocytoma cell lines (Bernard *et al.*, 1983). Rearrangement of c-*myc* has also been described in cell lines or fresh tissue from other lymphoid tumours, but these are less well characterised in terms of the precise genomic configuration (Rothberg *et al.*, 1984; Dalla-Favera *et al.*, 1983). Rearrangements of the c-*myc* proto-oncogene have been correlated with elevated expression but this does not appear to be invariably the case (Klein, 1981; Hamlyn & Rabbitts, 1983). In some Burkitt cell lines levels of c-*myc* mRNA comparable to those seen in normal cells are found, but the untranslocated allele is silent (Taub *et al.*, 1984). This raises the possibility that the translocation may result in expression at an inappropriate stage of cellular development. On the basis of this work, it has been suggested that abnormal expression of c-*myc* may be critically involved in development of lymphomas.

In contrast to the extensive study of Burkitt lymphoma derived cell lines there is little known about the expression of this gene in other types of lymphoid tumour or in normal lymphoid tissues. Elevated levels of c-*myc* mRNA have been found in some tumours (Rothberg *et al.*, 1984; Roy-Burnam *et al.*, 1983; Slamon *et al.*, 1984), but the interpretation of these results is complicated by the extensive cellular heterogeneity which may be present in malignant lymphomas.

We therefore decided to investigate c-*myc* expression in lymphoid tumours using monoclonal antibodies to the $p62^{c\text{-}myc}$ gene product. It was hoped that such an immunocytochemical study would give information about the cellular distribution of the c-*myc* product in both normal and malignant lymphoid tissue and the relationship of c-*myc* expression, if any, to cellular differentiation. It was hoped that it would be possible to comment on whether the gene was being aberrantly or constitutively expressed in tumour cells.

### Materials and methods

### *Tissue*

Tissue was obtained from 51 lymphoid tumours including most histological types commonly seen and 20 examples of normal lymphoid tissue (lymph node, spleen, thymus, tonsil and bone marrow). With the exception of two cases originating in East Africa all were recent biopsy specimens from

Correspondence: A.S. Jack.
Received 2 January 1986; and in revised form, 19 February 1986.

© The Macmillan Press Ltd., 1986

Glasgow Royal Infirmary. All tissue was fixed in buffered formol saline and embedded in paraffin wax.

The diagnosis was made on the basis of routine histology and a wide variety of immunocytochemical tests. The diagnostic categories broadly follow those described in the Kiel classification or the Lukes–Butler classification of Hodgkin's disease (Wright & Isaacson, 1983; Lukes & Butler, 1966).

*Monoclonal antibodies*

Two monoclonal antibodies were used. These were raised against synthetic peptides as previously described. The antibodies designated myc-1 6E10 and myc-1 9E10 have been shown to react with different regions of the p62$^{c\text{-}myc}$ molecule and to be monospecific on immunoblotting with various cell lines (Evan *et al.*, 1985). Myc-1 9E10 was in the form of a purified antibody (2 mg ml$^{-1}$) and was used as a dilution of 1/500 in Tris buffer pH 7.6. Myc-1 6E10 was in the form of hybridoma supernatant and was diluted to 1/20. These concentrations were obtained after titration studies on normal and neoplastic tissue.

*Immunocytochemistry*

Tissue sections were dewaxed and hydrated. All sections were routinely trypsinised for 5–15 min. Endogenous peroxidase activity was blocked using acidified methanol containing hydrogen peroxide. After washing in Tris saline, pH 7.6, sections were incubated overnight with diluted primary antibody at 20°C. The sections were washed and incubated for 30 min with optimally diluted rabbit anti-mouse-horseradish peroxidase conjugate (Dako). Antibody binding was demonstrated by immersion in di-aminobenzidine and hydrogen peroxide for 10 min. Sections were then washed, counterstained with haematoxylin and dehydrated. A control section was stained, for each specimen, omitting the primary antiserum.

### Results

*Antibody specificity*

An identical pattern of staining was obtained using both monoclonal antibodies. (Figure 1a, b). As these were raised to different parts of the p62$^{c\text{-}myc}$ molecule it renders fortuitous cross reactions, commonly seen with monoclonal antibodies, unlikely.

*Distribution of staining*

*Normal lymphoid tissue* All lymphoid cells seen in normal lymph node, spleen, tonsil and thymus were unstained with the exception of plasma cells (see Table I). (Figure 2). These cells showed marked and consistent cytoplasmic staining. In thymus strong granular cytoplasmic staining was present in the epithelial cells including Hassall's corpuscles. In tonsil granular cytoplasmic staining was present in crypt epithelium. In both spleen and lymph node rather faint cytoplasmic staining was seen in histiocytes. This was most marked in the tingeable body histiocytes associated with germinal centres.

Intense nuclear and cytoplasmic staining was present in the majority of haemopoietic precursor cells seen in normal bone marrow (Figure 1a, b). Weak cytoplasmic staining was sometimes seen in mature granulocytes.

*Lymphoid tumours* A summary of the staining patterns found in lymphoid tumours is given in Table II. A number of additional points should be noted. Two lymphoblastic tumours were included.

**Table I** C-*myc* in normal lymphoid and haemopoietic tissue

| *Tissue* | *Distribution of staining with anti*-p62$^{c\ myc}$ |
|---|---|
| Lymph node | (a) Granular cytoplasmic staining in some histocytes, especially those in germinal centres.<br>(b) Cytoplasmic staining of most plasma cells. All other lymphoid cells are negative. |
| Spleen | (a) Weak cytoplasmic staining in some sinus lining cells.<br>(b) White pulp negative except plasma cells. |
| Thymus | (a) Strong granular staining of epithelial elements.<br>(b) All lymphoid cells are negative. |
| Bone marrow | (a) Intense nuclear and cytoplasmic staining in all stages in differentiation.<br>(b) Weak cytoplasmic staining in mature granulocytes. |

**Figure 1** *Normal bone marrow.* (a) Monoclonal antibody 9E10; (b) monoclonal antibody 6E10; A similar distribution is seen with both antibodies. Nuclear and cytoplasmic immunostaining is present in myeloid and erythroid precursor cells and megakaryocytes. ( × 128)

**Table II** C-*myc* in lymphoid tumours

| *Tumour type* | *Number staining +ve* | *Comments* |
|---|---|---|
| B-lymphocytic/lymphoplasmacytic | 1/2 | Faint positive staining in some cells of the lymphoplasmacytic tumour. |
| B-lymphoblast (including BL) | 1/2 | Staining mainly cytoplasmic. |
| Follicle centre cell tumours | 3/17 | Positive staining very weak restricted to the cytoplasm of cells. |
| B-immunoblast | 0/1 | |
| T-lymphocytic | 0/2 | |
| T-lymphoblastic | 0/2 | |
| Pleomorphic peripheral T cell tumour | 2/6 | Positive staining (nuclear + cytoplasmic) restricted to immunoblast and multilobated elements. |
| T-immunoblastic | 5/5 | Variable cytoplasmic staining with occasional nuclei stained. |
| Hodgkin's disease | 7/12 | Positive staining present in variable numbers of Reed–Sternberg cells. |
| Plasma cell tumours | 2/2 | Cytoplasmic staining similar to normal plasma cells. |

One of these was a typical example of African Burkitt lymphoma (BL), which showed minimal staining of tumour cells but a moderate degree of staining of the substantial component of reactive histiocytes (Figure 3). The other case which differed morphologically from BL showed weak cytoplasmic staining. Positive staining was seen in all T-immunoblastic malignant lymphomas although in some cases this was rather weak. Some cells in these tumours showed nuclear as well as cytoplasmic staining.

In addition, large cell elements, presumably immunoblasts, were found to be stained in two cases of pleomorphic peripheral T cell tumours (Figure 4), although most of the small or intermediate sized cells were unstained (Figure 5). Two plasma cell tumours showed diffuse cytoplasmic and, occasionally nuclear staining of the type seen in normal plasma cells.

In the cases of Hodgkin's disease some Reed–Sternberg cells showed cytoplasmic and occasionally nuclear staining, although this was variable

**Figure 2** *Gastric mucosa.* This is a section of gastric mucosa who shows changes of chronic superficial gastritis. There is promient immunostaining of the cytoplasm of lamina propria plasma cells. The gastric surface epithelial cells are also positively stained. This was a feature of epithelial cells at all levels in the gastric glands. (×160)

**Figure 3** *Burkitt lymphoma.* There is staining of large inclusions within the cytoplasm of the reactive 'starry sky' histiocytes. Most of the tumour cells are unstained although a few cells show a very faint degree of positivity which is difficult to distinguish from background staining. (×120)

**Figure 4** *Pleomorphic T cell lymphoma.* This tumour contains a wide variety of types of neoplastic cell. The cells which are stained most prominently with the anti-p62$^{c-myc}$ are the large immunoblast-like cells. In these cells both the nucleus and cytoplasm are stained. (×160)

**Figure 5** *Pleomorphic T cell lymphoma in liver.* This section shows intense infiltration of a portal tract by neoplastic lymphoid cells. Cytoplasmic immunoreactivity is present in residual bile duct epithelium but there is no definite staining of tumour cells. (×160)

**Figure 6** *Follicle centre cell lymphoma in jejunum.* These sections show diffuse infiltration of the lamina propria of the jejunum by neoplastic lymphoid cells. Granular immunostaining is present localised to the cytoplasm of the intestinal glandular epithelium. The tumour cells are not stained. Similar staining was seen at all levels of the small intestinal crypt and villous epithelium. (×108)

even within individual cases. Positively staining Reed–Sternberg cells were seen in lymphocyte predominant nodular, nodular sclerosis and mixed cellularity subtypes of Hodgkin's disease.

*Normal non-lymphoid tissues* Non-lymphoid cells were often present in biopsies of lymphoid tissue. As shown in Table III, a wide variety of cell types showed immunoreactivity. Some cells, such as connective tissue cells, showed diffuse cytoplasmic staining, similar to that seen in plasma cells. In other cells, notably hepatocytes and jejunal epithelium, (Figure 6) p62$^{c-myc}$ was localised to coarse, intensely staining cytoplasmic granules. The perinuclear distribution of these granules in enterocytes suggested an association with the Golgi apparatus.

**Table III** C-*myc* in normal non-lymphoid tissues

| *Tissue* | *Distribution of staining with anti*-$p62^{c\text{-}myc}$ |
|---|---|
| Epidermis and other squamous epithelia | Granular cytoplasmic staining mainly in basal layer. |
| Small intestine | Granular perinuclear staining in both crypts and villous epithelium. |
| Liver | Granular cytoplasmic staining in hepatocytes and bile duct epithelium. |
| Salivary gland | Cytoplasmic staining in ducts. Salivary acini are negative. |
| Thyroid | Granular cytoplasmic staining in thyroid acinar cells. |
| Connective tissues | Cytoplasmic staining in both smooth and skeletal muscle. Weaker staining in other mesenchymal cells including adipocytes, fibroblasts and endothelium. |
| Stomach | Diffuse cytoplasmic staining of gastric antral epithelium. |

### Discussion

The aim of this study was to determine whether $p62^{c\text{-}myc}$ was detectable in normal lymphoid tissues and tumours by immunocytochemical methods and whether this was related to cellular differentiation. Twenty-one of 51 cases of malignant lymphoma showed some evidence of immunocytochemical reactivity with the anti $p62^{c\text{-}myc}$ monoclonal antibodies. However, in the majority of cases, especially follicle centre cell tumours, the intensity of staining was very weak relative to that seen in some normal tissues. Nevertheless some tentative conclusions can be drawn about the relationship of anti $p62^{c\text{-}myc}$ staining and cellular differentiation.

Positive staining was most consistent in T immunoblastic malignant lymphomas and this was further corroborated by the positive immunoreactivity in the immunoblast component of pleomorphic peripheral T cell tumours. It was of interest that many Reed–Sternberg cells in cases of Hodgkin's disease also stained positively; some authors hold the view that these cells are modified immunoblasts, in most cases of the T cell type (Stein *et al.*, 1984). It was not possible to determine whether normal T-immunoblasts contained $p62^{c\text{-}myc}$ since these cells are rarely seen in normal lymphoid tissue and suitable material from cases of reactive T immunoblastic hyperplasia such as infective mononucleosis was not available. Therefore it is impossible to comment on whether the tumour cells are aberrantly expressing the c-*myc* gene product.

Two cases of lymphoblastic malignant lymphoma were studied, one of which was a typical African BL. The latter case showed no definite evidence of tumour cell reactivity, but there was intense granular staining in the reactive histiocytes which are a prominent feature of this condition. The non-Burkett B-lymphoblastic malignant lymphoma showed weak cytoplasmic staining. Again no equivalent normal cell type could be studied as those cells are not identifiable in normal tissues.

In cultured lymphoid cells a transient increase in levels of c-*myc* mRNA is found after mitogen or growth factor stimulation at the $G_0/G_1$ transition (Kelly *et al.*, 1984). As transcription in some systems does not appear to vary during the cell cycle, regulation of c-*myc* mRNA may be partly related to variable rates of degradation (Blanchard *et al.*, 1985). In a wide range of other cells c-*myc* is induced in early $G_1$ phase and persists during S and $G_2$ phases of the cell cycle (Rabbitts *et al.*, 1985). Our results are not consistent with a simple relationship of $p62^{c\text{-}myc}$ expression and cell division. Positive immunoreactivity was seen in a wide range of cells, including terminally differentiated cells such as plasma cells and skeletal muscle cells as well as dividing cells such as haemopoietic marrow and $G_0$ arrested cells such as hepatocytes. Immunoreactive c-*myc* product has been described in normal testicular cells (Sikora *et al.*, 1985). An imperfect correlation with cell division and c-*myc* mRNA expression has also been found using *in situ* hybridisation of human embryos (Pheifer-Ohlsonn *et al.*, 1984). One possible explanation for the discrepancy between our results and the various *in vitro* studies could lie in the intracellular distribution of $p62^{c\text{-}myc}$. In cultured cells c-*myc* gene product is of short half life and localised to the nucleus (Rabbits *et al.*, 1985). In this study a nuclear distribution was seen in the actively dividing cells of bone marrow and in some tumours. However, in most normal cells which show immunoreactivity the localisation of $p62^{c\text{-}myc}$ was cytoplasmic. The pattern of staining of enterocytes and hepatocytes suggests that in these cells the protein was probably concentrated in the Golgi apparatus. This suggests that localisation of the protein, *in vivo,* may differ in cells which are dividing as opposed to those which are arrested or terminally differentiated.

In view of the unexpected results of this study the possibility of antibody cross-reactivity must obviously be considered. This is a serious problem when using single monoclonal antibodies. (Nigg *et al.*, 1982). However, in this study antibodies to separate parts of the $p62^{c\text{-}myc}$ were used and taken with the detailed characterisation of these antibodies, cross-reactivity would seem to be a highly unlikely explanation. The second problem lies in the sensitivity of the methods used. Immunohistochemical methods are known to be very sensitive but nevertheless it cannot be definitively stated that very low levels of $p62^{c\text{-}myc}$ may not be detected and that these could have biological significance. Tissues were fixed rapidly after removal from the patient which therefore makes it unlikely that c-*myc* protein would have been significantly catabolised before fixation. A further possibility is that the intracellular distribution of $p62^{c\text{-}myc}$ was altered by fixation, either by diffusion, or some other mechanism. However, it is difficult to envisage how the observed tissue-specific differences in distribution could be accounted for by this type of artefact. Furthermore, the intense granular localisation seen in some cells (Figures 5 and 6) would not be consistent with fixation associated diffusion.

In conclusion, our study provides little evidence to support the view that elevated expression of c-*myc* is a common or necessary feature of human malignant lymphomas. Widespread distribution of this protein in normal tissues raises a number of interesting questions concerning its function. These concern the possible differing functions of nuclear and cytoplasmic $p62^{c\text{-}myc}$ since it may be that one is active and the other a storage form of the molecule. It would also be relevant to ask whether c-*myc* is involved solely in the control of cell division or whether it participates in other aspects of cellular metabolism. These questions clearly merit further investigation. Finally, it does not appear that the immunocytochemical demonstration of $p62^{c\text{-}myc}$ is of value in the pathological diagnosis or subtyping of malignant lymphomas.

We would like to thank Dr A. Balmain and G.D. Binnie for helpful advice and Dr K. Sikora for facilitating this study. The manuscript was typed by Miss M. Habbick and the photographs were prepared by Mr T. Parker (Medical Illustration Department, Glasgow Royal Infirmary). A.S. Jack is a Wellcome Trust Lecturer.

### References

BERNARD, O., CORY, S., GERONDAKIS, S., WEBB, E. & ADAMS, J.M. (1983). Sequences of the murine and human cellular *myc* oncogene and two modes of *myc* transcription resulting from chromosome translocation in B lymphoid tumours. *EMBO J.*, **2**, 2375.

BLANCHARD, J.M., PIECHACZYK, M., DANI, C. & 4 others. (1985). C-*myc* gene is transcribed at high rate in $G_0$-arrested fibroblasts and in post-transcriptionally regulated in response to growth factors. *Nature*, **317**, 443.

DALLA-FAVERA, R., MARTINOTTI, S., GALLO, R.C., ERIKMAN, J. & CROCE, C.M. (1983). Translocation and rearrangements of the c-*myc* oncogene locus in human undifferentiated B-cell lymphomas. *Science*, **219**, 963.

ERIKSON, J., NISHIKURA, K., AR-RUSBIDI, A. & 5 others. (1983). Translocation of an immunoglobulin locus to a region 3′ of an unrearranged c-*myc* enhances c-*myc* transcription. *Proc. Natl Acad. Sci. USA.*, **80**, 581.

EVAN, G.I., LEWIS, G.K., RAMSAY, G. & BISHOP, J.M. (1985). Isolation of monoclonal antibodies specific for human c-*myc* proto-oncogene product. *Mol. Cell Biol.*, **5**, 3610.

GELLMANN, E.D., PSALLIDOPOULAS, M.C., PAPAS, T.S. & DALLA-FAVERA, R. (1983). Identification of reciprocal translocation sites within the c-*myc* oncogene and immunoglobulin $\mu$ locus in a Burkitt's lymphoma. *Nature*, **306**, 799.

HAMLYN, P.H. & RABBITTS, T.H. (1983). Translocation joins c-*myc* and immunoglobulin genes in Burkitt's lymphoma revealing a third exon on the c-*myc* oncogene. *Nature*, **304**, 135.

HAYDAY, A.C., GILLES, S.D., SAITO, H. & 4 others. (1984). Activation of a translocated human c-*myc* gene by an enhancer in the immunoglobulin heavy chain locus. *Nature*, **307**, 334.

KELLY, K., COCHRAN, B., STILES, C. & LEDER, P. (1984). The regulation of c-*myc* by growth signals. *Curr. Top. Microbiol. Immunol.*, **113**, 117.

KLEIN, G. (1981). The role of gene dosage and gene transpositions in carcinogenesis. *Nature*, **294**, 313.

LUKES, R.J. & BUTLER, J.J. (1966). The pathology and nomenclature of Hodgkin's disease. *Cancer Res.*, **26**, 1063.

NIGG, E.A., WATTER, E. & SINGER, S.J. (1982). On the nature of cross reactions observed with antibodies directed to defined epitopes. *Proc. Natl Acad. Sci. USA.*, **29**, 5939.

PAPAS, T.S., KAN, N.K., WATSON, D.K. & 5 others. (1984). *Myc*-related genes in viruses and cells. *Cancer cell.*, **2**, 153.

PHEIFER-OHLSONN, S., RYDNERT, J., GOUSTIN, A.S., LARSSON, E., BETSHOLTZ, C. & OHLSSON, R. (1984). Cell type-specific pattern of *myc* proto-oncogene expression in developing human embryos. *Proc. Natl Acad. Sci. USA.*, **82**, 5050.

RABBITTS, T.H., HAMLYN, P.H. & BAER, R. (1983). (1983). Altered nucleotide sequences of a translocated c-*myc* gene in Burkitt's lymphoma. *Nature*, **306**, 806.

RABBITTS, P.H., WATSON, J.V., LANAND, A. & 7 others. (1985). Metabolism of c-*myc* gene products with c-*myc* RNA and protein expression in the cell cycle. *EMBO J.*, **4**, 2009.

ROTHBERG, P.G., ERISMAN, M.D., DIEHL, R.E., ROVIGATTI, U.E. & ASTRIN, S.M. (1984). Structure and expression of the oncogene c-*myc* in fresh tumour material from patient with haemopoietic malignances. *Mol. Cell Biol.*, **4**, 1096.

ROY-BURMAN, P., DEVI, B.E. & PARKER, J.W. (1983). Differential expression of c-*erb*, c-*myc* and c-*myb* oncogene loci in human lymphomas and leukaemias. *Int. J. Cancer*, 32, 185.

SIKORA, K., EVAN, G., STEWART, J. & WATSON, J.V. (1985). Detection of the c-*myc* oncogene product in testicular cancer. *Br. J. Cancer*, **52**, 171.

SLAMON, D.J., DE KERNON, J.B., VERMA, I.M. & CLINE, M.J. (1984). Expression of cellular oncogene in human malignancies. *Science*, **224**, 256.

STEIN, H., LENNERT, K., FELLER, A.C. & MASON, D.Y. (1984). Immunohistological analysis of human lymphoma: Correlation of histological and immunological categories. *Adv. Cancer Res.*, **42**, 67.

TAUB, R., MOULDING, C., BATTEY, J. & 4 others. (1984). Activation and somatic mutation of the translocated c-*myc* gene in Burkitt's lymphoma cells. *Cell.*, **36**, 339.

WRIGHT, D.H. & ISAACSON, P.G. (1983). The biopsy pathology of the lymphoreticular system. p. 119. Chapman and Hall: London.